# LAMP2 regulates autophagy in the thymic epithelium and thymic stroma-dependent CD4 T cell development

**DOI:** 10.1080/15548627.2022.2074105

**Published:** 2022-05-19

**Authors:** Pedro M. Rodrigues, Laura G. Sousa, Chiara Perrod, Ana R. Maceiras, Pedro Ferreirinha, Rita Pombinho, Gema Romera-Cárdenas, María Gomez-Lazaro, Meryem Senkara, Jelena Pistolic, Didier Cabanes, Ludger Klein, Paul Saftig, Nuno L. Alves

**Affiliations:** a Instituto de Investigação e Inovação em Saúde, Universidade do Porto, Porto, Portugal; b Instituto de Biologia Molecular e Celular, Porto, Portugal; c Doctoral Program in Molecular and Cell Biology, Instituto de Ciências Biomédicas Abel Salazar, Universidade do Porto, Porto, Portugal; d Instituto de Engenharia Biomédica, Porto, Portugal; e Biochemisches Institut, Christian Albrechts-Universität Kiel, Kiel, Germany; f Genomics Core Facility, European Molecular Biology Laboratory, Heidelberg, Germany; g Faculty of Medicine, LMU Munich, Planegg-Martinsried, Institute for Immunology, Biomedical Center Munich, Munich, Germany

**Keywords:** Autophagy, LAMP2, TCR repertoire, thymic selection, thymus

## Abstract

Within the thymus, thymic epithelial cells (TECs) provide dedicated thymic stroma microenvironments for T cell development. Because TEC functionality is sensitive to aging and cytoablative therapies, unraveling the molecular elements that coordinate their thymopoietic role has fundamental and clinical implications. Particularly, the selection of CD4 T cells depends on interactions between TCRs expressed on T cell precursors and self-peptides:MHC II complexes presented by cortical TECs (cTECs). Although the macroautophagy/autophagy-lysosomal protein degradation pathway is implicated in CD4 T cell selection, the molecular mechanism that controls the generation of selecting MHC II ligands remains elusive. LAMP2 (lysosomal-associated membrane protein 2) is a well-recognized mediator of autolysosome (AL) maturation. We showed that LAMP2 is highly expressed in cTECs. Notably, genetic inactivation of *Lamp2* in thymic stromal cells specifically impaired the development of CD4 T cells that completed positive selection, without misdirecting MHC II-restricted cells into the CD8 lineage. Mechanistically, defects in autophagy in *lamp2*-deficient cTECs were linked to alterations in MHC II processing, which was associated with a marked reduction in CD4 TCR repertoire diversity selected within the *lamp2*-deficient thymic stroma. Together, our findings suggest that LAMP2 interconnects the autophagy-lysosomal axis and the processing of selecting self-peptides:MHC II complexes in cTECs, underling its implications for the generation of a broad CD4 TCR repertoire.

**Abbreviations**: AIRE: autoimmune regulator (autoimmune polyendocrinopathy candidiasis ectodermal dystrophy); AL: autolysosome; AP: autophagosome; Baf-A1: bafilomycin A_1_; B2M: beta-2 microglobulin; CTSL: cathepsin L; CD74/Ii: CD74 antigen (invariant polypeptide of major histocompatibility complex, class II antigen-associated); CFSE: carboxyfluorescein succinimidyl ester; CFU: colony-forming unit; CLIP: class II-associated invariant chain peptides; cTECs: cortical TECs dKO: double knockout; DN: double negative; DP: double positive; ENPEP/LY51: glutamyl aminopeptidase; FOXP3: forkhead box; P3 IFNG/IFNγ: interferon gamma; IKZF2/HELIOS: IKAROS family zinc finger 2; IL2RA/CD25: interleukin 2 receptor, alpha chain; KO: knockout; LAMP2: lysosomal-associated membrane protein 2; LIP: lymphopenia-induced proliferation; Lm: *Listeria monocytogenes;* MAP1LC3/LC3: microtubule-associated protein 1 light chain 3; MHC: major histocompatibility complex; mTECs: medullary TECs; PRSS16/TSSP: protease, serine 16 (thymus); SELL/CD62L: selectin, lymphocyte; SP: single positive; TCR: T cell receptor; TCRB: T cell receptor beta chain; TECs: thymic epithelial cells; UEA-1: *Ulex europaeus* agglutinin-1; WT: wild-type.

## Introduction

The thymus generates T cells expressing α β T cell receptors (TCRs) that recognize foreign antigens presented by self-major histocompatibility complex (MHC) molecules and are tolerant to self-components. This process is developmentally regulated by interactions between TCR expressed on T cell precursors (thymocytes) and self-peptide-MHC complexes presented by thymic epithelial cells (TECs) [1]. TECs express a myriad of soluble and cell-surface ligands that coordinate all stages of T cell differentiation. Importantly, the thymopoietic role of TECs is sensitive to aging and cytoablative therapies, which compromise T-cell responses to pathogens and vaccination in the elderly and immunocompromised patients [2]. Therefore, the identification of novel regulators of TEC functionality is vital to comprehend the foundations of T cell-immunity and to restore thymopoiesis in disorders associated with defective T cell responses. TECs are divided into functionally distinct cortical (cTECs) and medullary (mTECs) subtypes. While cTECs guide T cell lineage commitment and positive selection, mTECs regulate negative selection and T regulatory cell differentiation [3]. The functional segregation between cTECs and mTECs is, in part, determined by their unique antigen processing and presentation properties, which are controlled by specific protein degradation machineries and transcriptional programs [4,5]. However, how these proteolytic machineries coordinate the presentation of selective self-peptide-MHC complexes remains elusive at the molecular level.

In this regard, mTECs employ conventional proteolytic pathways to generate “public” MHC I- and MHC II-bound peptides that negatively select autoreactive thymocytes or drive their deviation into regulatory T cells [4]. These key steps for tolerance induction depend on the distinctive ability of mTECs to express tissue-restricted antigens, a process that is mediated by AIRE and FEZ family zinc finger 2 [1]. Alternatively, cTECs harbor unique antigen processing properties that generate sets of “private” MHC I- and MHC II-bound self-peptides, which are critical for the positive selection of double-positive (DP) thymocytes and their commitment into CD4 or CD8 T cells. The generation of MHC I-bound peptides that select CD8^+^ T cells depends on the distinct proteolytic activity of PSMB11/β5t (proteasome (prosome, macropain) subunit, beta type 11)-containing thymoproteasome [5,6]. For the generation of MHC II-bound peptides, cTECs employ unconventional endogenous MHC II-loading pathways that involve the autophagy-mediated transport of cytosolic proteins into lysosomes [7,8]. Lysosomal proteases, including CTSL (cathepsin L) and PRSS16/TSSP (protease, serine 16 (thymus)) contribute to the generation of specific self-peptides that select CD4^+^ T cells [9–12]. Although autophagy has been coupled to the control of thymic selection [7,8,13], the molecular mechanism that links this catabolic pathway to the generation of selective self-peptides in the lysosomes remains elusive. Our study identifies a role for LAMP2 (lysosomal-associated membrane protein 2) in the thymic stroma as a determinant regulator of CD4 T cell development and TCR repertoire formation.

## Results

### LAMP2 is dispensable for the establishment of major TEC microenvironments

**Figure 1.**
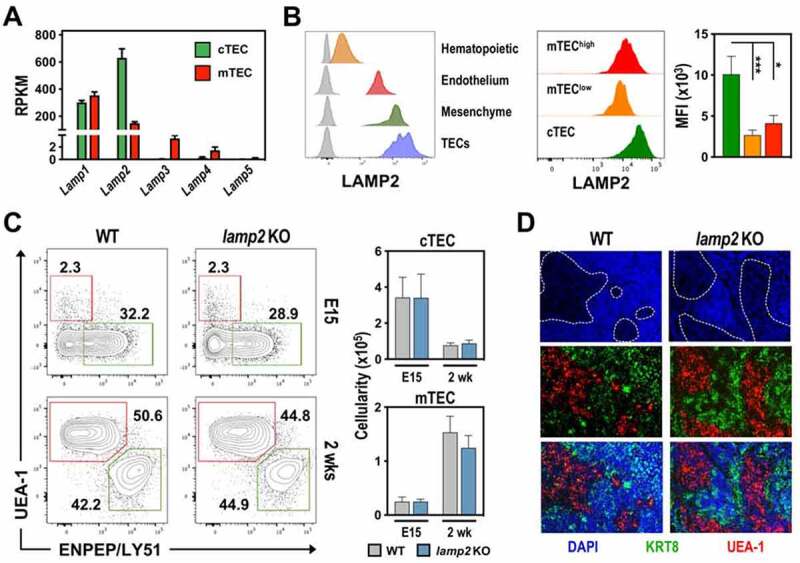
The deficiency in *Lamp2* does not affect global cTEC/mTEC differentiation. (A) Expression (RNAseq analysis) of LAMP family members in cTECs (green) and mTECs (red) from WT mice. Error bars represent mean ± SEM. (B) LAMP2 expression in distinct thymic cell populations in 2-week-old WT and *lamp2* KO mice. Left histogram: Hematopoietic (PTPRC/CD45^+^), endothelial (PECAM1/CD31^+^), mesenchymal (PDGFRA-PDGFRB [αβ]^+^) and TEC (EPCAM^+^) cells (n = 3), (WT and *lamp2* KO subsets in color and gray, respectively); Right histogram: cTECs (ENPEP/Ly51^+^ UEA-1^−^), mTEC^lo^ (ENPEP/Ly51^−^ UEA-1^+^ CD80^lo^) and mTEC^hi^ (ENPEP/Ly51^−^ UEA-1^+^ CD80^hi^). Bars graphs represent mean fluorescence intensity (MFI) of LAMP2 expression. Data representative of 3 independent experiments (n = 12 animals). (C) cTEC and mTEC composition in embryonic day 15.5 (E15.5) and 2-week-old WT and *lamp2* KO thymus. Dot plots show a representative ENPEP/Ly51 and UEA staining and graphs represent the average cellularity of c/mTECs in the indicated timepoints from 3 independent experiments (n = 11–14 animals/group). (D) Immunofluorescence analysis of thymic sections from 2-week-old WT and *lamp2* KO thymus stained for DAPI (blue), UEA-1 (red) and KRT8 (keratin 8; green). Results in **A-C** are shown as mean ± SEM. * *p* < 0.05; ** *p* < 0.01; *** *p* < 0.001.

To identify new candidates involved in the antigen processing capacity of cTECs, we analyzed our transcriptomic data from postnatal cTECs and mTECs [14], together with available data sets from other studies [15,16]. Given that MHC II-bound peptide generation involves the endosomal–lysosomal pathway, we sought genes associated with these vesicular compartments that were selectively enriched in cTECs relatively to mTECs. *Ctsl* (cathepsin L) and *Prss16* were expectedly among the top genes upregulated in cTECs. We selected to analyze LAMP2, which was the only member of the LAMP family upregulated (4-fold) in cTECs (Figure 1(A)**, Figure S1A**). LAMP2 is a lysosomal membrane protein that regulates macroautophagy and chaperone-mediated autophagy (CMA) [17], promoting the fusion of autophagic vacuoles with lysosomes [18,19]. LAMP2 has been also implicated in the regulation of MHC II presentation in human B cells [20,21]. Yet, its involvement in T cell development and selection is unknown.

In man, genetic mutations in *LAMP2* are associated with Danon Disease, a fatal X-linked syndrome caused by lysosomal dysfunction [22]. Mice deficient for *Lamp2* (*lamp2* knockout; *lamp2* KO) recapitulate key aspects of the human condition, including the accumulation of autophagic vacuoles in several cell types [18,19,23]. Despite the increased mortality in early adulthood, *lamp2* KO mice survive the first weeks of life [19]. Hence, to study the function of LAMP2 in the establishment of major TEC subsets, we analyzed the *lamp2* KO thymus during embryonic and postnatal life. We began by analyzing the expression of LAMP2 protein in embryonic wild-type (WT) TECs, and using cells from *lamp2* KO mice as a control for the antibody staining (**Figure S1B**). Within the postnatal thymus, TECs represented the highest LAMP2-expressing subset, relatively to thymocytes and other non-TEC stromal cells, including endothelial and mesenchymal cells. The corresponding *lamp2* KO subsets were included to further validate antibody staining. In line with the transcriptomic data, cTECs significantly expressed higher levels of LAMP2 relatively to mTEC^lo/hi^ subtypes (Figure 1(B)). The deficiency in *Lamp2* did not alter the generation of primordial cTECs and mTECs at embryonic day 15 nor affected the cellularity of the main cTEC and mTEC subsets in the 2-week-old thymus (Figure 1(C)). Further analysis of the expression of cTEC-associated markers (LY75/CD205, CD40) or mTEC subpopulations, including mTEC^lo^ (MHCII^lo^ CD40^lo^ CD80^lo^), mTEC^hi^ (MHCII^hi^ CD40^hi^ CD80^hi^), CCL21^+^ and AIRE^+^ cells, revealed no differences between WT and *lamp2* KO TECs (**Figure S1C**). Additionally, the spatial organization of cortical and medullary areas was normal in the *lamp2* KO thymus (Figure 1(D)). Our results indicate that LAMP2 is not essential for the establishment of global cTEC and mTEC compartments.

### LAMP2 in thymic stromal cells specifically controls the development of CD4 T cells

The pleiotropic defects of *lamp2* KO mice and the broader expression of LAMP2 in several tissues hampered a direct assessment of its role in TEC-mediated T cell selection. To circumvent this aspect, we employed a well-established thymic transplantation model wherein WT or *lamp2* KO embryonic thymi were grafted under the kidney capsule of nude adult recipients, referred to hereafter as WT-Nude and *lamp2* KO-Nude. In these chimeras, the deficiency in *Lamp2* was restricted to TECs, endothelial and mesenchymal cells, allowing the assessment of how recipient-derived T cell precursors (from WT origin) developed and were selected within WT and *lamp2* KO thymic stroma, in the absence of confounding effects of *lamp2*-deficiency in other tissues. Although TECs have a non-redundant role in T cell commitment and selection [4], this approach did not formally exclude a potential contribution for LAMP2 in endothelial and mesenchymal cells in these processes.

**Figure 2.**
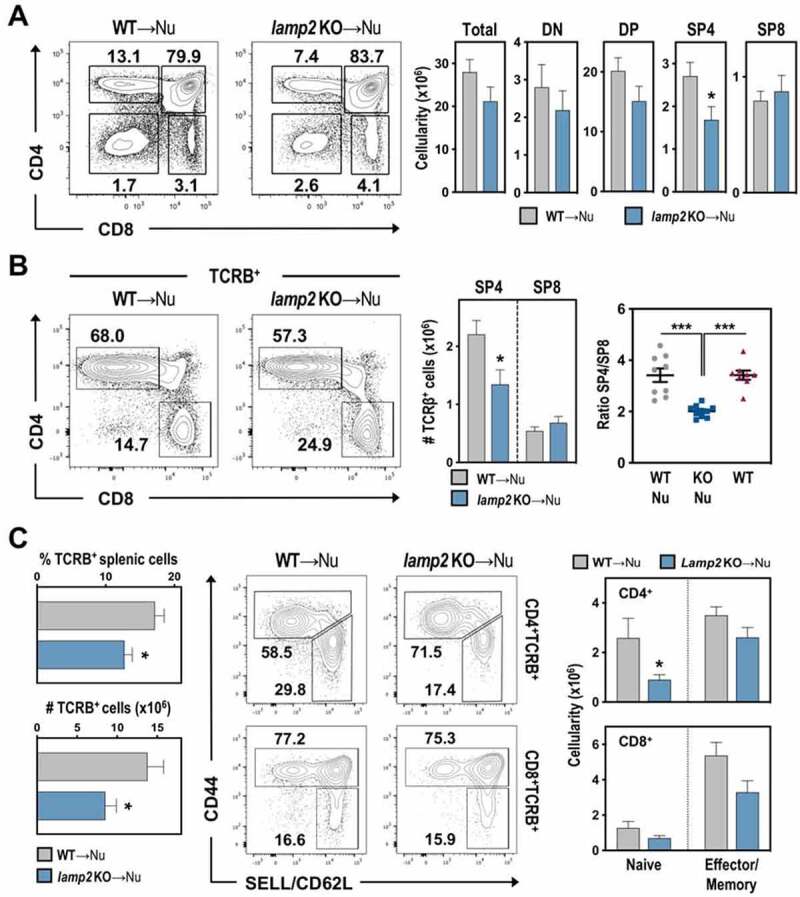
T cell development in WT and *lamp2* KO thymic grafts. Representative dot plots of CD4 and CD8 expression within (A) total thymocytes and (B) TCRB^+^ thymocytes in thymic grafts of Nude recipients transplanted with WT (WT-Nu) and *lamp2* KO (*lamp2* KO-Nu) ectopic thymus, 8–10 weeks after transplantation. Numbers on the plots indicate the frequencies of the different subsets. Bar graphs in A and B depict the average absolute cellularity of the thymus and the indicated thymocyte subsets in WT-Nu (gray) and *lamp2* KO-Nu (blue). In B, ratio of the frequency of SP4:SP8 is shown in WT-Nu and *lamp2* KO-Nu, as well in the endogenous thymus. (C) Analysis of peripheral T cells in spleens from WT-Nu and *lamp2* KO-Nu mice. Graphs (left) represent the average % and numbers of TCRB^+^ T cells in the spleen. Dot plots show CD44 and SELL/CD62L expression within CD4 and CD8 T cells. Graphs (right) show average cellularity of naïve (SELL/CD62L^+^ CD44^lo^) and effector/memory (SELL/CD62L^±^ CD44^hi^) CD4 and CD8 T cells. Data represent an average of 3 independent experiments (n = 10 WT and *lamp2* KO ectopic thymi). Results in **A-C** are shown as mean ± SEM. * *p* < 0.05; ** *p* < 0.01; *** *p* < 0.001.

Analysis of T cell development 8–10 weeks post-transplantation showed that the percentages of immature double-negative (DN) and DP thymocytes were similar in WT-Nude and *lamp2* KO-Nude thymic grafts. While the numbers of total, DN and DP thymocytes appeared slightly reduced, these differences were not statistically significant (Figure 2(A)**, Figure S2A**), indicating that LAMP2 is dispensable for β–selection and DN-DP transition. Notably, the frequencies of single-positive 4 (SP4) and SP8 thymocytes were altered in *lamp2* KO thymi. These changes resulted in a specific and statistically significant, reduction in the number of SP4 but not SP8 cells (Figure 2(A)). Analysis of T cells expressing high levels of TCRB (T cell receptor beta chain) (TCRB^hi^) confirmed the decrease of circa 40% in the average number of SP4 cells in the *lamp2* KO thymus (1.3x10^6^ cells) relatively to the cellularity found in the WT counterparts (2.2x10^6^ cells). These changes led to a deviation in SP4:SP8 ratios in the mutant thymi as compared to controls, and endogenous thymi of WT mice (Figure 2(B)). The thymic alterations coincided with changes in the peripheral T-cells, with a statistically significant reduction in the number of *lamp2* KO thymus-derived splenic naïve CD4^+^ T cells. The numbers of effector/memory CD4, and naïve and effector/memory CD8 T cells were moderately reduced, but these changes were not statistically significant (Figure 2(C)). Together, these results pointed to an impaired CD4 T cell development within the *lamp2*-deficient thymic microenvironment.

**Figure 3.**
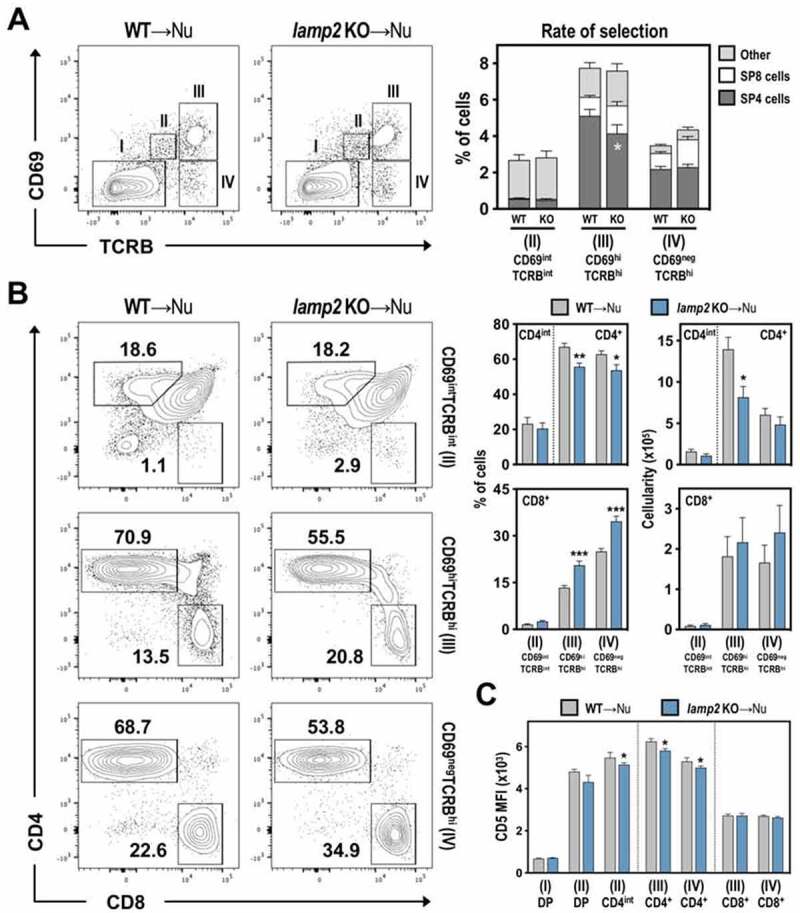
Pre- and post- positive T cell selection in WT and *lamp2* KO thymic grafts. (A) Flow cytometry analysis of pre- and post- positive selection in WT (WT-Nu) and *lamp2* KO (*lamp2* KO-Nu) ectopic thymus grafted into Nude recipients. Dot plots show the analysis of CD69 and TCRB expression on total thymocytes from WT-Nu and *lamp2* KO-Nu ectopic thymus. Stage I (TCRB^neg/int^ CD69^neg^), II (TCRB^int^ CD69^int^), III (TCRB^hi^ CD69^hi^) and IV (TCRB^hi^ CD69^neg^) defined pre-, recently-, post-selected and mature stages, respectively. Graph represents the average percentages of total populations II, III and IV. In each bar (populations II, III and IV bar) is shown the corresponding frequency of SP4 and SP8 cells, which were calculated based on the frequencies of SP4 and SP8 shown in panel B. (B) CD4/CD8 expression within stages II (TCRB^int^ CD69^int^), III (TCRB^hi^ CD69^hi^) and IV (TCRB^hi^ CD69^neg^), which were defined on total thymocytes (A). Graphs represent the average percentages and absolute numbers of the indicated thymocyte subsets in WT-Nu (gray) and *lamp2* KO-Nu (blue). (C) Graphs represent the MFI of CD5 expression in the indicated subsets (defined as shown in Figure S2). Data shown represent an average of 10 ectopic WT and 10 ectopic *lamp2* KO thymi from 3 independent experiments. Results in **A-C** are shown as mean ± SEM. * *p* < 0.05; ** *p* < 0.01; *** *p* < 0.001.

To identify the stage at which CD4^+^ T cell development was altered in *lamp2* KO thymus, we examined distinct stages of pre- and post-positive selection, based on the differential expression of TCRB and CD69 expression on thymocytes: Population I (TCRB^neg/int^ CD69^neg^) contains mostly pre-selected DP thymocytes; Population II (TCRB^int^ CD69^int^) harbors TCR-signaled cells initiating positive selection; Population III (TCRB^hi^ CD69^hi^) represents post-positively selected SP thymocytes; Population IV (TCRB^hi^ CD69^neg^) consists of more mature SP cells [24]. Although the frequency of populations II, III and IV within total thymocytes were similar, we found a noticeable alteration in the frequency of SP4 and SP8 in post-positively selected cells (TCRB^hi^ CD69^hi^, III) of the *lamp2* KO-Nude thymi (Figure 3(A-B)). This led to a significant reduction in the numbers of post-positively selected SP4, but not SP8 thymocytes, in *lamp2* KO-Nude thymi (Figure 3(B)). We further analyzed the expression of CD5 in thymocyte subsets as this correlates with TCR affinity for self-peptide-MHC ligand [25,26]. Stage II DP thymocytes from WT and *lamp2* KO thymus expressed comparable CD5 levels, suggesting normal TCR signaling during the early stages of positive selection (Figure 3(C)**, Figure S2B**). Early after positive selection, DP thymocytes transverse through a CD4^+^CD8^int^ (CD4^int^) transitional stage prior to their commitment into CD4 and CD8 lineage [27]. The frequency and numbers of stage II CD4^int^ cells in *lamp2* KO-Nude mice were comparable to the WT-Nude setting (Figure 3(B)). Yet, the levels of CD5 in stage II CD4^int^ and III–IV SP4 thymocytes were moderately reduced within the *lamp2* KO-Nude thymus, suggesting an attenuation of TCR signaling in cells that completed positive selection (Figure 3(C)**, Figure S2B**). Additionally, stage IV SP4 thymocytes did not accumulate in the *lamp2* KO thymus nor presented elevated CD5 levels (Figure 3(B-C)**, Figure S2B**), arguing against excessive TCR signaling. To study whether *lamp2*-deficiency could affect other steps of CD4 T cell development that depend on mTECs, we analyzed SP4 maturation and the regulatory T cell differentiation [1]. The proportion of immature (CD24^+^ SELL/CD62L^−^) and mature (CD24^−^ SELL/CD62L^+^) SP4 thymocytes were comparable within WT and *lamp2* KO thymus. Additionally, the frequency of immature (IL2RA/CD25^+^ Forkhead box P3 (FOXP3)^−^ and IL2RA/CD25^−^ FOXP3^+^) and mature CD4 T regulatory (IL2RA/CD25^+^ FOXP3^+^) cells was similar in WT and *lamp2* KO thymi, suggesting that the maturation of T reg was not affected by *lamp2*-deficiency. Although moderately reduced, the numbers of mature CD4 T regulatory cells were not statistically different (**Figure S2C-D**). Contrarily, the cellularity of immature (IL2RA/CD25^+^ FOXP3^−^) T reg, immature and mature SP4 was statistically diminished in *lamp2* KO thymus. The reduction in these subsets may arise from the reduction in SP4 that completed positive selection, which was the subset mostly affected by the deficiency in *Lamp2* and included immature T reg and SP. Together, our results suggested a more prominent function for LAMP2 in regulating the development of CD4^+^ T cells that complete positive selection.

**Figure 4.**
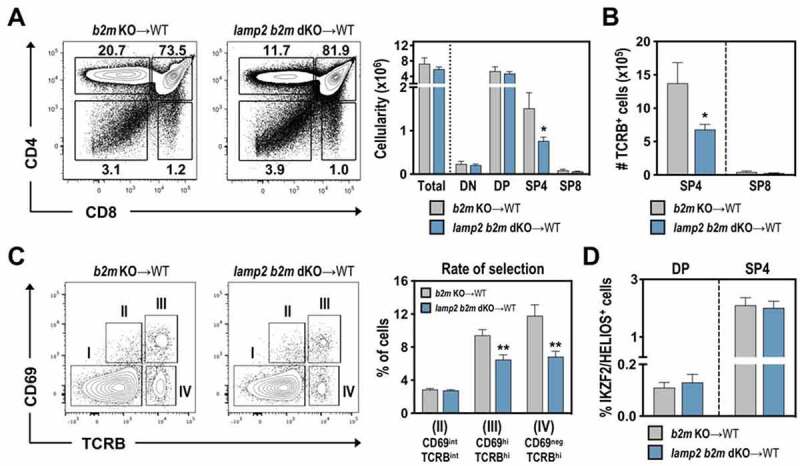
Combined deficiency in *Lamp2* and *B2m* specifically reduces CD4 T cell development, without redirecting MHC II-restricted cells into the CD8 lineage. (A-B) Flow cytometry analysis of T cell development in *b2m* KO (*b2m* KO-WT) and in *lamp2* and *b2m* double-KO (*lamp2 b2m* dKO-WT) thymus grafted into WT recipients. (A) Representative dot plots of CD4 and CD8 expression on total thymocytes, with numbers indicating the frequencies of the different subsets. Graphs represent the cellularity of total thymocytes and the indicated subsets in *b2m* KO (gray) and *lamp2 b2m* dKO (blue). (B) Graph represents the absolute numbers of SP4 and SP8 within mature TCRB^+^ thymocytes. Data shown represent an average of 9 *b2m* KO and 10 *lamp2 b2m* dKO ectopic thymi from 3 independent experiments. (C) Dot plots show the flow cytometric profile for CD69 and TCRB on total thymocytes from *b2m* KO-WT and *lamp2 b2m* dKO-WT ectopic thymi. Stage I (TCRB^neg/int^ CD69^neg^), II (TCRB^int^ CD69^int^), III (TCRB^hi^ CD69^hi^) and IV (TCRB^hi^ CD69^lo/neg^) were defined as represented. Graphs represent the average percentages of cells in the indicated gates in *b2m* KO-WT (gray) and *lamp2 b2m* dKO-WT (blue) ectopic thymi. (D) Graphs represent the average frequency of IKZF2/HELIOS^+^ cells in DP and SP4 from *b2m* KO-WT (gray) and *lamp2 b2m* dKO-WT (blue) (defined as shown in Figure S3B). Data include an average of 2 independent experiments (n = 6 *b2m* KO and 8 *lamp2 b2m* dKO-WT). Results in **A-D** are shown as mean ± SEM. * *p* < 0.05; ** *p* < 0.01; *** *p* < 0.001.

The selection of CD4^+^ T cells depends on “strong” and persistent TCR-mediated signals [28]. The decreased CD5 levels on SP4 cells could suggest that the reduction in SP4 cells in *lamp2* KO thymus was functionally linked to a defective CD4 commitment. In this scenario, MHC II-restricted thymocytes were misdirected toward the CD8 lineage due to an attenuated TCR signaling. To examine this possibility and prevent MHC-I-driven CD8 selection, we generated mice double deficient for *Lamp2* and *B2m* (beta-2 microglobulin) (*lamp2 b2m* dKO mice). Thymic transplantations were set with *b2m* KO or *lamp2 b2m* dKO embryonic thymus grafted into WT mice. Owing to the deficiency in *b2m*, and consequently MHC-I expression, the selection of MHC-I-restricted SP8 thymocytes was expectedly abolished in *b2m* KO thymic grafts [29], so that mature TCRB^+^ cells mostly comprised SP4 thymocytes (Figure 4(A-B)). Notably, the frequency and numbers of SP4 thymocytes were reduced in *lamp2 b2m* dKO thymus, without a concomitant increase in SP8 thymocytes (Figure 4(A-B)**, Figure S3A**). Moreover, recently post-selected (TCRB^hi^ CD69^hi^) and mature (TCRB^hi^ CD69^neg^) SP4 cells were significantly reduced in frequency and numbers in *lamp2 b2m* dKO thymic grafts (Figure 4(C)**, Figure S3A**). To evaluate the possible contribution of LAMP2 in cortical and medullary negative selection at a polyclonal level, we analyzed the expression of IKZF2/HELIOS (IKAROS family zinc finger 2) on DP (TCRB^lo/int^ IL2RA/CD25^−^ FOXP3^−^) and CD4SP (TCRB^hi^ IL2RA/CD25^−^ FOXP3^−^) thymocytes [26]. The frequency of IKZF2/HELIOS^+^ cells was similar in both WT- and *lamp2* KO-derived thymocyte subsets (Figure 4(D)**, Figure S3B**). These observations support that deficiency in *Lamp2* does not redirect MHC II-restricted cells into CD8 lineage, but instead prevents the development of CD4 T cells that completed positive selection.

### Alterations in autophagy in lamp2 KO cTECs are associated with changes in MHC II processing

**Figure 5.**
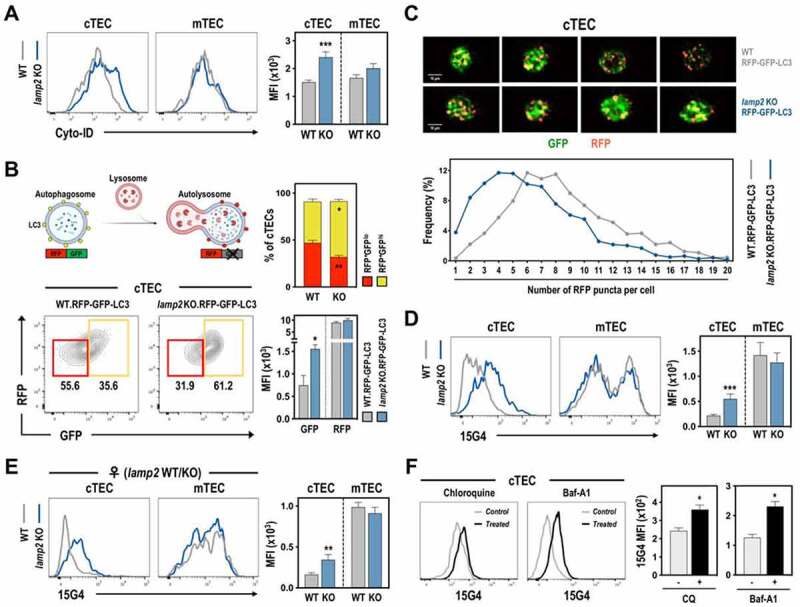
Defective autophagy and MHC II processing in *lamp2* KO cTECs. (A-B) Autophagic flux in cTECs from 12 days old WT and *lamp2* KO mice was analyzed by flow cytometry. (A) Histograms show representative Cyto-ID analysis in cTEC and mTEC from WT and *lamp2* KO. Graphs represent the MFI of Cyto-ID in the indicated subsets. (B) Scheme (top left) represents autophagic flux using RFP-GFP-LC3 mice. Differential detection of GFP and RFP allows the distinction between AP (GFP^+^ RFP^+^) and AL (RFP^+^). Dot plot (bottom left) represents GFP and RFP expression in cTECs of WT and *lamp2* KO RFP-GFP-LC3 mice. Graphs (top right) represent the average frequency of RFP^+^ GFP^hi^ (yellow) and RFP^+^ GFP^low^ (red) cells in WT and *lamp2* KO RFP-GFP-LC3 cTECs. Graphs (bottom right) represent the MFI of GFP and RFP expression in WT (gray) and *lamp2* KO (blue) RFP-GFP-LC3. (C) WT and *lamp2* KO RFP-GFP-LC3 cTECs (EPCAM^+^ ENPEP/LY51^+^) were analyzed by imaging flow cytometry. Representative images are shown. Graph show the distribution of the number of RFP puncta in WT (n = 1345 cells) and *lamp2* KO (n = 1422 cells) RFP-GFP-LC3 cTECs. Data in **A-C** include an average of 2–3 independent experiments (n = 4–6 WT and n = 4–6 *lamp2* KO. (D) Flow cytometry analysis of 15G4 staining on cell surface of cTECs (ENPEP/LY51^+^ UEA-1^−^) and mTECs (ENPEP/LY51^−^UEA-1^+^) from 12 days old WT and *lamp2* KO mice. Histograms show representative staining and graphs represent the MFI of 15G4 staining in the indicated subsets. Data include an average of 4 independent experiments (n = 6 WT and 7 *lamp2* KO). (E) Histograms show representative 15G4 staining on the cell surface of WT and *lamp2* KO c/mTECs from *lamp2* WT/KO heterozygous mice. Graphs represent the MFI found in indicated subsets in control (gray) and mutant (blue) cells (n = 8 from 4 experiments). (F) Postnatal day 3–5 WT thymus were treated overnight with chloroquine (50 μM) or with Baf-A1 (0.5 μM) for 5 h. Histograms show the representative staining with 15G4 staining in control (light gray) and chloroquine-treated (black) or Baf-A1-treated (black) in WT cTECs. Graphs represent the MFI in control (gray) and treated (dark gray/ black) cTEC (n = 4–6 animals from 3–4 independent experiments). Results in **A-F** are shown as mean ± SEM. * *p* < 0.05; ** *p* < 0.01; *** *p* < 0.001.

To determine whether the defect in CD4 T cell development could be mechanistically linked to changes in autophagy provoked by the deficiency in *Lamp2*, we evaluated the autophagic flux in TECs. *Ex vivo* isolated *lamp2* KO cTECs displayed an increased level of Cyto-ID, a tracer that marks AP and AL [30] (Figure 5(A)), indicative of altered autophagy dynamic. Moreover, *lamp2* KO mice were crossed with transgenic mice that ubiquitously expressed a RFP-GFP-MAP1LC3A/LC3 in a pH-dependent manner (RFP-GFP-LC3). In RFP-GFP-LC3 mice, RFP is stable and GFP is quenched in the acidic pH of lysosomal vesicles, allowing the distinction between AP (GFP^+^ RFP^+^) and AL (RFP^+^) [31]. The RFP-GFP-LC3 expression was predominantly detected in TECs relatively to the hematopoietic and non-TEC stroma of WT and *lamp2* KO thymus (**Figure S4A**). Moreover, the proportion of RFP^+^-GFP^+^-LC3 TECs was specifically increased in *lamp2* KO cTECs, but not mTECs (Figure 5(B) and **Figure S4B**). The accumulation of RFP^+^-GFP^+^-LC3 cells resulted from an augmented expression of GFP-LC3, but not RFP-LC3, in *lamp2* KO cTECs (Figure 5(B)). We further analyzed the autophagic flux at the single-cell level by employing imaging flow cytometry. *lamp2* KO cTECs contained cells with a reduced number of RFP^+^-LC3 puncta/cell compared to WT counterparts, being the *lamp2* KO subset mostly composed of cells with dual RFP^+^-GFP^+^-LC3 puncta (Figure 5(C)). As the status of autophagy flux correlates with the number of autophagic vesicles, these results indicated that AP-lysosome fusion was markedly affected in *lamp2* KO cTECs, as reported in other cells deficient in *Lamp2* [18,19,23].

We next studied whether changes in LAMP2-mediated autophagy were associated with alterations in self-peptide:MHC II processing in cTECs, which in turn could condition their capacity to select CD4 T cells. MHC II molecules are stabilized in the endoplasmatic reticulum by their binding to the CD74 antigen/Ii (invariant polypeptide of major histocompatibility complex, class II antigen-associated). Subsequently, CD74/Ii is degraded into a fragment called CLIP (class II-associated invariant chain peptides) in the MIIC (MHC II processing compartment) to permit antigen-binding [32,33]. To evaluate MHC II processing and maturation, we employed 15G4 antibody that recognizes I-A^b^ occupied by the CD74/Ii degradation intermediates small leupeptin induced protein or CLIP [34,35]. Strikingly, 15G4 staining was substantially increased in *lamp2* KO cTECs, but not in mTECs, relative to WT counterparts (Figure 5(D)). In line with previous studies [10], mTECs displayed higher 15G4 staining as compared to cTECs (Figure 5(D)). Control and mutant cTECs, as well as mTECs, expressed comparable levels of MHC II and I molecules (**Figure S4C**), suggesting a specific role of LAMP2 in the processing of MHC II, but not in its trafficking to the cell membrane. Exploring the fact that *Lamp2* resides on X chromosome, we analyzed TECs from *lamp2* WT/KO heterozygous thymus, which due to random X–inactivation in females included WT and *lamp2* KO TECs (**Figure S4D**). *lamp2* KO cTECs presented an augmented 15G4 staining, even within a microenvironment containing WT cells, suggesting that LAMP2 controls MHC II processing in a cell-autonomous manner (Figure 5(E)). Given the role of CTSL in the degradation of CD74/Ii in cTECs [9,10], we studied if altered MHC II processing resulted from a decrease in CTSL or lysosomal functions. The proteolytic function of CTSL and global lysosomal activity were increased in *lamp2* KO cTECs (**Figure S4E-F**). These results suggest that the augmented levels of MHC II-bound to CD74/Ii degradation intermediates did not result from a decrease in CTSL activity or lysosomal processing capacities. Lastly, we evaluated whether changes in LAMP2-mediated autophagy could be functionally linked to the alterations in MHC II processing. To do so, postnatal day 3–5 WT thymi were treated with two inhibitors, chloroquine and bafilomycin A_1_ (Baf-A1), of AP-lysosome fusion. Interestingly, chloroquine and Baf-A1 treatment increased 15G4 staining in WT cTECs (Figure 5(F)). The elevated 15G4 staining of WT mTECs was not substantially altered in Baf-A1-treated cells (**Figure S4G**). Worth noting, mTECs are less abundant in the postnatal thymus [14] and were sensitive to apoptosis following culture. Although the results with pharmacological inhibitors should be considered with caution due to additional specific or off-target effects, our data suggest that inhibition of AP-lysosome fusion in WT cTECs mimicked the phenotype observed on *lamp2* KO cTECs, and establish a presumable causality between changes in autophagy flux and MHC II processing.

### LAMP2 in thymic stroma controls the generation of a broad CD4 TCR repertoire

**Figure 6.**
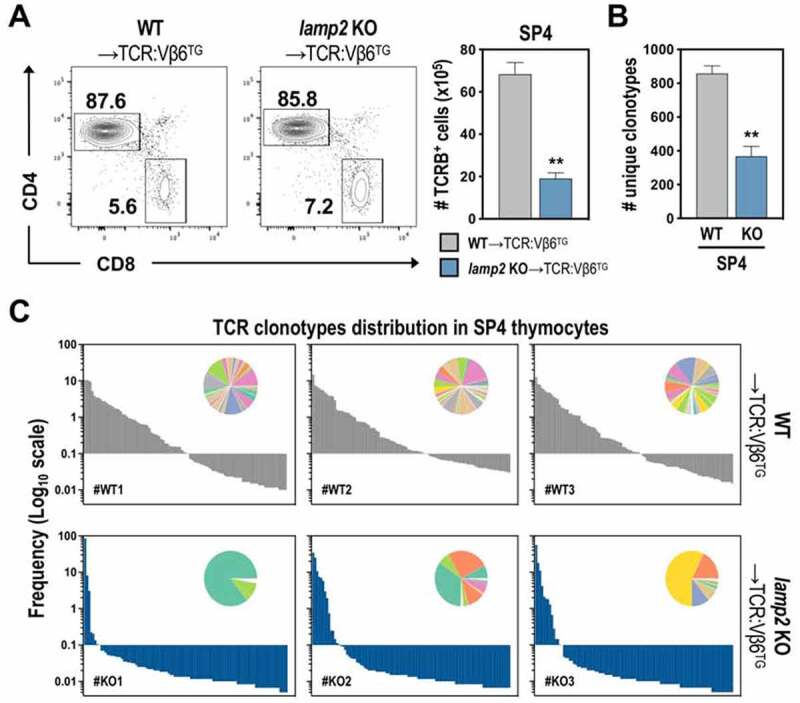
Analysis of SP4^+^ TCR repertoire in WT and *lamp2* KO thymus. (A) T cell development in WT (WT-TCR:Vβ6^TG^) and *lamp2* KO (*lamp2* KO-TCR:Vβ6^TG^) ectopic thymus grafted into fixed TCRB/TCRβ^TG^ recipients. CD4 and CD8 expression within mature TCRB^+^ thymocytes. Graphs represent the absolute number of the indicated thymocyte subsets in WT-TCR:Vβ6^TG^ (gray) and *lamp2* KO-TCR:Vβ6^TG^ (blue) thymi. Data shown represent an average of 2 independent experiments (n = 6 WT and 6 *lamp2* KO). Graphs indicated (B) the number and (C) frequency of unique sequences of *Tcr* clones found in SP4 thymocytes developing within WT and *lamp2* KO ectopic thymus. Pie graphs in C depict a colorimetric representation of the most abundant clonotypes found in each thymi. Results in **A-B** are shown as mean ± SEM. * *p* < 0.05; ** *p* < 0.01; *** *p* < 0.001.

Lastly, we determined whether thymic stroma deficient in *Lamp2* altered the generation of TCR repertoire diversity in developing CD4 T cells, possibly by affecting the MHC II-processing and the generation of positive selecting self-peptides in cTECs. Despite considerable efforts to identify the nature of self-peptides that promote positive selection [5], it remains challenging to conduct this biochemical characterization with sensitivity in rare populations, such as TECs. Due to this limitation, we determined the TCR clonal composition of SP4 thymocytes generated in WT or *lamp2* KO thymi. The assessment of TCR diversity could serve as an indirect indicator of the quality of the MHC II-peptide ligandome presented by *lamp2* KO cTECs. Given the vast combinations of TCRA/α-TCRBβ chains in SP4 thymocytes in a broad polyclonal setting, we used mice expressing a fixed transgenic TCR Vβ6 (beta6) chain [36] as recipients of thymic grafts. This model referred as TCR:Vβ6Tg, reduced the complexity of the TCR repertoire to the diversity of TCRA chains and favored CD4^+^ T cell selection, as confirmed by the accumulation of SP4 thymocytes in WT thymic grafts (Figure 6(A)**, Figure S5A**). The bias toward CD4 lineage may result from the fact that the Vβ6 chain is derived from a MHC II-restricted TCR [36]. Consistent with the results in the fully polyclonal TCR repertoire of Nude and WT recipients, *lamp2*-deficiency restrained the number of SP4 TCR:Vβ6Tg thymocytes (Figure 6(A)). The cellularity of DP cells was also reduced in TCR:Vβ6Tg model (**Figure S5A)**, possibly due to the ubiquitous expression of MHC-II-restricted Vβ6 chain that may anticipate the effects provoked by *lamp2-*deficiency in the DP stage. For repertoire analysis, we isolated RNA from 3 samples of 1.10^6^ (FASC sorted) SP4 (CD4^+^ TCRB^+^) thymocytes from WT and *lamp2* KO thymus. The *Tcra* gene was specifically amplified and the generated libraries were subjected to deep sequencing analysis of *Tcra* transcripts. We recovered 0.7–1,2.10^6^ sequences in SP4 cells derived from WT and *lamp2* KO grafts, from which 0.6–2.10^5^ corresponded to specific TCRs (**Figure S5B**, *first two columns*). Further analysis identified 1,3–1,5.10^3^ unique TCRs in the WT setting. This number (0,3–0,4.10^3^) was notably reduced in SP4 cells from *lamp2* KO thymi (**Figure S5B**, *third column*). Given the variable number of retrieved TCR sequences, the analysis was normalized to 6.10^5^ randomized TCR sequences/sample. This threshold was defined by the lower number of sequences identified on one sample (KO2) (**Figure S5B**). Following unbiased normalization, the number of unique clonotypes remained significantly lower in *lamp2* KO-derived SP4 thymocytes relatively to the ones generated from WT thymus (Figure 6(B)**, Figure S5B**). Additionally, the distribution of TCRA sequences was distinctively different. While clonotypes of WT-derived thymus presented the stereotypical broad distribution expected to be found in a polyclonal repertoire, *lamp2* KO counterparts displayed a clear skewing in the TCR catalog (Figure 6(C)). Further analysis revealed that the clonality index was markedly increased in mutant samples (**Figure S5C**).

These results led us to evaluate whether the restricted thymic SP4 TCR repertoire affected the function of peripheral T cells generated within *lamp2* KO thymic stroma. To do so, we resorted to the fully polyclonal TCR repertoire model wherein WT and *lamp2* KO thymi were grafted in Nude recipients, as described in Figure 2. We probed the proliferative response of splenic CD4 and CD8 T cells of WT- or *lamp2* KO-nu mice to polyclonal TCR stimulation *in vitro*, and found no differences between groups as measured by CFSE analysis (**Figure S6A**). These results suggest that CD4 and CD8 T cells developing in *lamp2* KO thymic stroma respond to a polyclonal TCR-mediated stimulation. Lastly, we determined whether thymic defects in TCR repertoire formation influence the outcome of an immune response to a live intracellular pathogen. We employed a well-defined model of T-cell dependent recall response to *Listeria monocytogenes* (*Lm*) [37]. Recipient mice were inoculated with 2.10^3^ CFU (colony-forming units) of *Lm* at 12 weeks post thymic transplantation, to guarantee peripheral T cell reconstitution, and then rechallenged with 1.10^6^ CFU 4 weeks later (**Figure S6B**). Non-thymic grafted nude recipients did not resist the infection protocol (**Figure S6C**), highlighting the requirement for a T cell-mediated response in this model. We assessed the T cell reconstitution in the spleen, and the clearance of *Lm* in the liver of infected WT-nu and *lamp2* KO-nu animals 3 days after the secondary challenge. Although reduced, the numbers of naïve and effector/memory splenic CD4 and CD8 T cells were not significantly altered in WT-nu or *lamp2* KO-nu (**Figure S6D)**. All recipient mice were reconstituted. Despite the variability in CFU counts and the absence of complete bacterial clearance in both genotypes, the average CFU counts were slightly elevated in *lamp2* KO-nu mice, which presented an increase in the number of animals with a higher bacterial burden (≥ log_10_4) (**Figure S6E**). Interestingly, the CFU counts inversely correlated with the amount of IFNG/IFNγ (interferon gamma)-producing CD4 found in *lamp2* KO-nude after the recall response. These cells have been shown to provide protection against *Lm* [37]. A similar association was observed in IFNG/IFNγ-producing CD8, but this correlation was not significant (**Figure S6E**). Although antigen-specific responses were not monitored, *lamp2* KO-nu appeared to present a compromised *in vivo* T-cell response to secondary infection by *Lm*. Together, our results suggest that thymic stroma deficient in *Lamp2* restricts not only the quantity of selected CD4^+^ T cells, but also the quality of their TCR repertoire.

## Discussion

Following the discoveries on the contributions of CTSL and PRSS16/TSSP to the generation of selecting MHC II:peptides complexes [9–12], there have been few advances in the mechanism that regulates CD4^+^ T cell selection. Our findings suggest that LAMP2 may represent a functional liaison that interconnects autophagy and processing of self-peptides-MHC II for selection in cTECs, which in turn regulates the development of CD4 T cells and the diversity of their TCR repertoire. LAMP2 is expressed in TECs, but it was also detected in mesenchymal and endothelial cells of the thymic stroma. As non-TEC subsets do not have a major role in thymocyte selection [4], it is possible to conjecture that the defects in CD4 T cell development in *lamp2* KO thymic stroma may arise from LAMP2-dependent failures in TECs. Future studies with TEC-specific conditional knockout mice will provide more precise details on the cell-intrinsic role of LAMP2 in TECs. The strength and duration of the interaction between TCR and MHC II:self-peptide ligandome have implications in thymic selection [27,28]. Thymic stroma deficient in *Lamp2* led to a particular reduction in CD4 T cells that completed positive selection. While changes in TCRB^int^ CD69^int^ and TCRB^hi^ CD69^hi^ suggest defects in positive selection, alterations in TCRB^hi^ CD69^hi^ and TCRB^hi^ CD69^neg^ may reflect survival defects following positive selection, altered lineage commitment or negative selection. Our findings indicate that the development of CD4 T cells was not mechanistically associated with an abnormal redirection of MHC II-restricted cells into CD8 T cell lineage. Moreover, the reduced CD5 levels in TCRB^int^ CD69^int^ and TCRB^hi^ CD69^hi^ SP4s and the lack of alterations in IKZF2/HELIOS in DP and SP4 collectively suggested that TCR signaling and negative selection of SP4 thymocytes were not augmented. Instead, they may support the hypothesis that LAMP2 regulates the development of MHC II-restricted CD4 T cells that completed positive selection in the thymic cortex.

The observation that deficiency in *Lamp2* particularly affected autophagy flux in cTECs, but not mTECs nor other non-TEC thymic stromal cells, are in line with the reported high levels of autophagy in cTECs [7,8,38,39] and the role of LAMP2 in lysosome-AP fusion [18,19]. Moreover, autophagic vesicles gain access to the MHC II compartment in TECs [40] and autophagy has been linked with the generation of the peptide repertoire presented by MHC II [7,8,38,39]. We found that CTSL and lysosomal activities were elevated in *lamp2* KO cTECs, arguing against the possibility that alterations in CD74/Ii-MHC II processing results from a failure in its proteolytic function. The increased lysosomal activity was also reported in the hippocampus of *lamp2* KO mice [41], suggesting that LAMP2-mediated autophagy impacts lysosomal function. Several non-mutually exclusive possibilities might explain the reduction in SP4 selection by *lamp2*-deficient thymic stroma. The inhibition of AP-lysosome fusion in *lamp2* KO cTECs can compromise the shuttling of autophagy-derived cytoplasmatic selective antigens into the late endosomal MIIC loading pathway. Under these conditions, CTSL [9,10], PRSS16/TSSP [11,12] and other lysosomal proteases may not have access to substrates to produce MHC II-restricted self-peptides specialized for positive selection of CD4 T cells. This can lead to a compensatory upregulation of lysosomal activities as typically reported in models of lysosomal dysfunction or lysosomal storage disorders [18]. Additionally, the alteration in autophagic flux might also disturb CD74/Ii-MHC II trafficking to MIIC compartment or H2M function, thereby preventing the complete degradation of CD74/Ii or self-peptide loading. Furthermore, CTSL also contributes to the generation of MHC II–bound self-peptides presented by cTECs [9,10,35]. Thus, the increase in CTSL activity in *lamp2* KO cTECs could also interfere with the quality of MCH II-peptide ligandome presented by these cells during positive selection. Hence, the incomplete degradation of CD74/Ii-MHC II in *lamp2* KO cTECs may result from altered trafficking of CD74/Ii-MHCII to MIIC and/or from a reduction in MHC II-loaded with positive selection-inducing self-peptides, due to an altered antigen processing and peptide repertoire generation. The consequent change in the composition of selective MHC II ligands may lead to a reduction of CD4 T cells that are completing positive selection and a skewing of their TCR repertoire. It will be important to elucidate the biochemical mechanism by which LAMP2 potentially regulates MHC class II-peptide generation in cTECs. Under regular conditions, endogenous antigens may access MHC II molecules through several LAMP2-regulated autophagic rotes [17,18]. Future studies should also aim at investigating whether LAMP2-dependent macroautophagy delivers cytosolic antigens into autolysosomes for processing before loading to MHC II, and/or antigens are directly routed into the lysosome via CMA. In this regard, ATG5-dependent autophagy has been linked to positive selection of CD4 T cells [8,39], but it also contributes to mTEC-mediated tolerance induction [38,42]. Worth noting, *lamp2*-deficiency did not cause major defects in mTEC-dependent function nor disturbed tolerance, as measured by the presence of autoantibodies/lymphocytic infiltrates at 12 weeks post-transplantation (data not shown). Future studies should address whether breaks in tolerance can unfold in aged settings. Alternatively, autophagy may have distinct roles in c/mTEC function, whereby the specificity of the route and its consequence in thymic selection, can also be imposed by distinct downstream elements. While ATG5 participates in early phases of autophagy and may have a broader effect in cTEC- and mTEC-mediated thymic selection [8,39], LAMP2 mostly regulates later stages of the autophagic process and MHC II processing in cTECs. Future analysis should examine whether changes provoked by *lamp2*-deficiency in cTECs account for a more dedicated role in the control of CD4 T cell positive selection.

The observations that *lamp2*-deficiency in thymic stroma induced a skewing, but not a complete block, in CD4 TCR repertoire diversity, suggests that LAMP2 may regulate the generation of particular sets of autophagy-dependent MHC II-bound positive selecting peptides in cTECs. This hypothesis is in line with previous studies [43] supporting that positive selection is limited by the bioavailability of specific self-peptide/MHC ligands expressed by cTECs. Still, previous studies showed that a single MHC-peptide ligand can positively select multiple TCRs [44,45], indicating that positive selection is relatively permissive regarding the composition of MHC-peptide ligands in cTECs. Assuming that LAMP2 contributes to the generation of particular peptide:MHC II ligands, its deficiency may have variable effects depending on the plasticity of a given TCR. Future analysis employing TCR transgenics will allow defining LAMP2-dependent specificities and the contribution of LAMP2 in positive and negative selection. Additionally, post-selected thymocytes expressing high-affinity TCR intrathymically proliferate in response to their self-peptide/MHC ligands [46]. Thus, the dominance of particular clonotypes within the *lamp2*-deficient thymus may result from the expansion of SP4 T cells bearing favorable TCR affinities for overrepresented self-peptide/MHC ligands. Importantly, the TCR repertoire analysis was conducted in total SP4 cells, without segregating conventional and regulatory CD4 T cells. Although T reg comprises a small fraction of thymic SP4, one can only presume that their TCR repertoire is also affected. Future analysis is required to clarify the impact of LAMP2 in TCR repertoire formation and the function of T regulatory cells. Lastly, the defect in thymic production imposed by *lamp2*-deficiency impacted on the peripheral T cell pool, mostly on the numbers of naïve CD4 T cells, and to a less extent on naïve CD8 T cells and effector/memory CD4 and CD8 T cells. In the thymic transplant of Nude mice, the establishment of the peripheral T cell pool depends on thymic output and LIP (lymphopenia-induced proliferation). As thymic CD8 T cell development was not affected, the reduced peripheral CD8 T cell pool may arise indirectly from insufficient CD4 T cell help. In this regard, it was shown that the LIP of CD8 T cells depends on CD4 T cells [47–49]. Additionally, our results indicate that CD4 and CD8 T cells, which developed in *lamp2*-deficient thymic stroma, respond to polyclonal TCR-mediated signaling. Yet, *lamp2* KO-nude recipients appear to hold a compromised T cell-dependent recall immune response to *Lm* infection. Future studies should clarify the peripheral consequences of these thymic defects, including a more in-depth analysis of T cell homeostasis, antigen-specific responses and tolerance, as well as the role of CD4 T cell in B cell responses and CD8 T cell memory.

In sum, our findings suggest that *lamp2*-deficiency in thymic stroma conditions the development and TCR repertoire formation of CD4 T cells. These findings provide a novel framework to further investigate how LAMP2 functionally interconnects autophagy and lysosomal generation of selecting self-peptides in cTEC, and its implications for CD4 T cell positive selection. Moreover, our results may also open new avenues for the identification of the “peptidic-self” in the thymus, which remains one of the crucial gaps in our understanding of thymus biology.

## Material and methods

### Mice

We used *lamp2* KO, *b2m* KO, RFP-GFP-LC3 Tg, TCR:Vβ6 Tg and Nude mice that have been described previously and are all in a C57BL/6 background [19,29,31,36]. Mice were housed under specific pathogen–free conditions, and all animal experiments were performed in accordance with European guidelines (Directive 2010/63/EU). For fetal studies, the day of the vaginal plug detection was designated as embryonic day (E) 0.5.

#### Thymic kidney transplants

Thymi from WT and *lamp2* KO mice were transplanted under the kidney capsule of recipient mice as described [50]. Briefly, thymic lobes from E15.5 WT and *lamp2* KO mice were cultured for 2 days with 1.35 mM 2’- deoxyguanosine (SIGMA, D0901) to deplete lymphoid cells. Prior to transplantation recipient mice were pre-treated with the analgesic buprenorphine (0.05 mg/kg of body weight) and anesthetized with a gaseous mixture of 3% isofluorane and oxygen.

### TEC and hematopoietic cell isolation

TECs were isolated as described [14,50,51]. Hematopoietic cells from thymus and spleen were prepared by mechanical disruption of the respective tissues. Splenic red blood cells were lysed using erythrocyte lysis solution: 155 mM ammonium chloride (Sigma-Aldrich, A9434), 10 mM potassium bicarbonate (Sigma-Aldrich, 237,205).

### Flow cytometry

Cells were pre-treated with FC block (anti-FCGR3/CD16-FCGR2B/CD32 antibodies TruStain fcX; Biologend, Biolegend, 101,320). Cell suspensions were stained as described [19–21] with Alexa Fluor 488/FITC-conjugated anti-CD8A (clone 53–6.7; eBioscience, 11–0081-82), anti-CD44 (clone IM7; Biolegend, 103,022), anti-15G4 (Santa Cruz Biotechnology, sc-53,946), anti-CD5 (clone 53–7.3; Biolegend, 100,605), anti-PECAM1/CD31 (clone MEC13.3; Biolegend, 102,514) and anti-CCL21 (clone 59,106; R&D Systems, IC457G-100UG); PE-conjugated anti-CD4 (clone GK1.5; eBioscience, 12–0041-82), anti-SELL/CD62L (clone MEL-14; eBioscience, 12–0621-81), anti-TCRB (clone H57-597; eBioscience, 12–5961-82), anti-CD80 (clone 16–10A1; eBioscience, 12–0801-82), anti-ENPEP/LY51 (clone 6C3; eBioscience, 12–5891-82), anti-LAMP2 (clone M3/84; Biolegend, 108,505), anti-CD40 (clone 3/23; BD Pharmingen, 553,791) and anti-MHCI^Db^ (clone 28–14-8; eBioscience, 12–5999-82); PerCP-Cy5.5-conjugated anti-PTPRC/CD45.2 (clone 104; Biolegend, 109,828), anti-TCRB (clone H57-597; eBioscience, 45–5961-80) and anti-MHCI^Kb^ (clone AF6-88.5; Biolegend, 116,515); PerCP-eFluor710 anti-CD4 (clone GK1.5; eBioscience, 46–0041, 82); PE-Cy7-conjugated anti-IL2R/CD25 (clone PC.61.5; eBioscience, 25–0251-82) and anti-CD69 (clone H1.2F3; eBioscience, 25–0691-81); APC/eFluor660-conjugated anti-CD8 (clone 53–6.7; eBioscience, 50–0081-82), anti-CD80 (clone 16–10A1; eBioscience, 17–0801-82), anti-EPCAM (clone G8.8; Biolegend, 118,212), anti-CD40 (clone 1C10; eBioscience, 17–0401-81) and anti-CD74 (In1/CD74, Biolegend, 151,003); APC-eFluor780-conjugated anti-I-A/I-E (clone M5/114-15-2; eBioscience, 47–5321-82); eFluor450-conjugated anti-CD24 (clone M1/69; eBioscience, 48–0242-82); BV421 conjugated anti-EPCAM (clone G8.8; Biolegend, 118,225) and anti-CD69 (clone H1.2F3; Biolegend, 104,527); BV510 conjugated anti-CD24 (clone M1/69; Biolegend, 101,831); BV605 conjugated anti-SELL/CD62L (clone MEL-14; Biolegend, 104,437); anti-PDGFRA (clone APA5; Biolegend, 135,916); BV650 conjugated anti-CD80 (clone 16–10A1; Biolegend, 104,731) and anti-CD8A/CD8α (clone 53–6.7; Biolegend, 100,741); BV785 conjugated anti-CD4 (clone GK1.5; Biolegend, 100,453). The binding of biotinylated *Ulex europaeus* agglutinin-1 (UEA-1) (Vector Laboratories, B-1065-2), anti-ENPEP/LY51 (clone 6C3; BD Pharmingen, 553,159), anti-LY75/CD205 (clone NLDC-145; Biolegend, 138,211), and anti-PDGFRB (clone APB5; Biolegend, 136,009) were revealed by either PE-Cy7-conjugated (eBioscience, 25–4317.82) or BV711-conjugated (Biolegend, 405,241) streptavidin. For intracellular staining, cells were prepared according to the supplier’s protocol (FOXP3 staining kit, eBioscience, 00–5523-00) and stained with FITC-conjugated anti-IKZF2/HELIOS (clone 22F6; Biolegend, 137,204), APC/eFluor660-conjugated anti- FOXP3 (clone FJK-16s; eBioscience, 2,059,207) and anti-Aire (clone 5H12; eBioscience, 1,929,296) antibodies. Flow cytometry was performed on a LSRFortessa, with data analyzed on FlowJo software (BD). Cell sorting was performed on a FACSAria I (BD), with purities >96%.

### Immunohistological analysis

Thymi were prepared for immunofluorescence as described [14]. Briefly, samples were fixed in 4% paraformaldehyde (Electron Microscopy Sciences), and 8-μm sections were stained with biotinylated-UEA-1 (Vector Laboratories, B-1065-2) and anti-rat KRT8/cytokeratin 8 (Troma-1; DSHB, AB 531826) as primary antibodies, with Alexa Fluor 647 anti-rat (Invitrogen, A21247) and streptavidin Alexa Fluor 555 (Invitrogen, S21381) as secondary antibodies. Vectashield mounting medium with DAPI (Vector Laboratories, H-1200-10) was used to prepare the slides. Analysis was performed in an AxioImager.Z1 (Zeiss). Images were processed with and Fiji Software.

### Autophagy analysis

We stained cells with Cyto-ID autophagy detection kit (Enzo, ENZ-51031-0050) according to the manufacturer’s protocol. In brief, cells were first washed with PBS, mixed with Cyto-ID staining solution and incubated at 37°C for 30 min (Cyto-ID) and subjected to flow cytometry analysis. To block autophagy, thymi were cultured with chloroquine (50 μM; Enzo, ENZ-51031-0050) for 16 h and Baf-A1 (0.5 μM; SIGMA, B1793) for 5 h.

### CTSL and lysosomal activity assays

CTSL and lysosome activities were respectively monitored using Magic Red CTSL (ImmunoChemistry Technologies, 941) and Lysosome-Specific (Biovision, K448) detection kits, according to the manufacture’s protocol. Briefly, cells were either incubated with a magic red staining solution containing the CTSL fluorogenic substrate or Lysosome-Specific Self-Quenched Substrate for 1 h at 37°C in DMEM (10% FBS). Cells were then washed and stained with TEC markers for flow cytometry analysis. In cells with active CTSL, the substrate fluoresces red upon proteolytic cleavage. The fluorescent signal is proportional to the intracellular lysosomal activity in cells with active lysosomes.

### TCR sequencing

SP4 thymocytes (1.0 × 10^6^ cells) derived from TCR:Vβ6Tg hematopoietic progenitors and developing in WT or *lamp2* KO thymus grafts were purified by cell sorting. RNA was extracted using RNeasy Mini kit (Qiagen, 74,106) following the manufacturer’s instructions. Full-cDNA library was prepared using Mint-2 kit (Evrogen, SK005), which introduces 5′-adapters to cDNA fragments, according to the manufacturer’s instructions. The *Tcra* gene was then specifically amplified using Platinum^TM^ *Taq* DNA polymerase high fidelity (Invitrogen, 11,304,011) and a primer pair specific for the 5′-adapter and the C region of the *Tcra* gene. The sequencing library was prepared using the Nextera kit (Illumina, FC-131-1024), in which each sample was barcoded, and sequenced using 250 bp paired-end illumina MiSeq technology. High-throughput sequencing were performed at Gene Core facility (EMBL, Germany).

### Deep sequencing data analysis

Paired-end 250 bp illumina sequencing data were initially trimmed using Trimmomatic and subsequently merged using PEAR. clonotypeR toolkit was then used to perform TCR sequence annotation and quantitative analysis in R [52–54]. Out of the X raw reads obtained from the 6 samples (3 WT and 3 *lamp2* KO), we identified X-Y TCR clonotypes from X-Z productive TCR sequences. For the samples to be comparable, the analyses were performed on 60,000 randomly selected TCR sequences for each data set as it was the lowest number of TCR sequences found in a data set. The presented clonality metric is 1− Pielou’s evenness index and can vary from 0 to 1 (more diverse to less diverse). The Pielou’s evenness corresponds to the Shannon’s entropy (using log 2) for each sample divided by the number of unique clonotypes (in log 2) of the same sample [54,55].

### In vitro T cell activation

WT and *lamp2* KO-derived CD4 and CD8 T cells were sorted, labeled with 1 μM of CellTrace^TM^ CFSE (carboxyfluorescein diacetate succinimidyl ester; ThermoFisher Scientific, C34554) and cultured in the presence of 1 μg/ml anti-CD3 mAb (clone 145–2C11; BD Pharmingen, 553,057) and 5 μg/ml anti-C28 mAb (clone 37.51; BD Pharmingen, 553,294). The precursor frequency of dividing cells (percentage of cells in the initial population that undergone one or more divisions after culture) was calculated as follows: [∑n ≥ 1(Pn/2 n)]/[∑n ≥ 0(Pn/2 n)], where n is the division number that cells have gone through and Pn is the number of cells in division, as described [56].

### Listeria infection

Mice were inoculated with *Lm* reference strain EGDe as described in [57]. Thymic grafted Nude recipients were intravenously infected 12 weeks post-transplantation, through the tail vein with 2.10^3^ colony-forming units (CFUs) of *Lm*. Mice were rechallenged with 1.10^6^ CFUs of *Lm* 28 days after the first priming. Three days after secondary infection, mice were sacrificed, livers were aseptically removed, homogenized in PBS, and homogenates were serially diluted and plated on BHI-agar (Fisher Scientific, 11,708,872). *Lm* colonies were enumerated after 24 h incubation at 37°C. For the intracellular IFNG/IFN-γ analysis, total spleen cells were stimulated for 5 h at 37°C, using the Cell Activation Cocktail, containing phorbol 12-myristate 13-acetate, ionomycin and brefeldin A (Biolegend, 423,304) according to the manufacturer’s instructions. After cell surface staining, cells were prepared according to the supplier’s protocol (FOXP3 staining kit; eBioscience, 00–5523-00), stained with PE-labeled anti-IFNG/IFNγ Ab (clone XMG1.2; Biolegend, 505,807) and analyzed by flow cytometry.

### Statistical analysis

Analysis was performed using Prism 9.1.1 software (GraphPad Software). The two-tailed Mann-Whitney test was used for statistical differences between groups. For multiple comparisons, a two-way ANOVA was used. *p* < 0.05 was considered significant.

## Supplementary Material

Supplemental Material

## Availability of data and materials:

Data was deposited in the GEO database (PRJNA752538).
