# Supplementary material for: LAMP2 regulates autophagy in the thymic epithelium and thymic stroma-dependent CD4 T cell development
Source: Autophagy. 2022 May 19;19(2):426–39. doi: 10.1080/15548627.2022.2074105 (PMC9851248; doi:10.1080/15548627.2022.2074105)
Supplement: Supplemental Material [file KAUP_A_2074105_SM0071.zip › Supplemental Information_Rodrigues_KAUP_2022_0061R5.docx]

**Supplemental Information:**

**LAMP2 regulates autophagy in the thymic epithelium and thymic stroma-dependent CD4 T cell development**

Pedro M. Rodrigues^1,2*^, Laura G. Sousa^1,2,3*^, Chiara Perrod^1,2*^, Ana R. Maceiras^1^, Pedro Ferreirinha^1,2^, Rita Pombinho^1,2^, Gema Romera-Cárdenas^1,2^, María Gomez-Lazaro^1,4^, Meryem Senkara^5^, Jelena Pistolic^6^, Didier Cabanes^1,2^, Ludger Klein^7^, Paul Saftig^5^ and Nuno L. Alves^1,2^.

^1^ Instituto de Investigação e Inovação em Saúde, Universidade do Porto, Portugal.

^2^ Instituto de Biologia Molecular e Celular, Porto, Portugal.

^3^ Doctoral Program in Molecular and Cell Biology, Instituto de Ciências Biomédicas Abel Salazar, Universidade do Porto, Porto, Portugal.

^4^ Instituto de Engenharia Biomédica, Porto, Portugal

^5^ Biochemisches Institut, Christian Albrechts-Universität Kiel, Kiel, Germany.

^6^ Genomics Core Facility, European Molecular Biology Laboratory, Heidelberg, Germany.

^7^ Institute for Immunology, Biomedical Center Munich, Faculty of Medicine, LMU Munich, Planegg-Martinsried, Germany.

**Supplementary figures and legends:**

**Figure S1.** LAMP2 expression in the thymus. (**A**) The gene list shows the top, most highly expressed genes that are associated with the gene ontology term “lysosomes”, obtained from RNA sequencing analysis of cTECs and mTEC of postnatal thymus [21-25]. cTEC/mTEC columns represent fold change. (**B**) LAMP2 expression (flow cytometry) in TECs (PTPRC/CD45^-^ EPCAM^+^) from embryonic day 14.5 WT and *lamp2* KO mice. (**C**) Analysis of TECs from WT and *lamp2* KO thymus from 2-week-old mice for a broad panel of cTEC- and mTEC-specific markers. Top row: Histograms show the expression of CD40, LY75/CD205 and MHC II in WT and *lamp2* KO cTECs. Dot plots show the expression of CD80 and MHC II (2^nd^ row), CD80 and CD40 (3^rd^ row) and AIRE and CCL21 (bottom row) in WT and *lamp2* KO mTECs. Graphs represent the average cellularity of respective mTEC subsets (mTEC^lo/hi^, CCL21^+^ and AIRE^+^). Results in **C** are shown as mean ± SEM.

**Figure S2.** Thymic T cell development in WT- and *lamp2* KO-Nude mice. (**A**) Flow cytometry analysis of early stages of T cell development in thymic grafts of Nude recipients transplanted with WT (WT-Nu) and *lamp2* KO (*lamp2* KO-Nu) ectopic thymus, 8-10 weeks after thymic transplantation. Representative plots of CD44 and IL2RA/CD25 expression on DN thymocytes (gated on Lin^-^ cells). ETPs are defined as Lin^-^CD177^+^CD44^+^IL2RA/CD25^-^. (**B**) Histograms show representative CD5 expression in DPs from TCRB^neg^ CD69^neg^ (Stage I), DPs and CD4^int^ from TCRB^int^ CD69^int^ (stage II), SP4 and SP8 from TCRB^hi^ CD69^hi^ (stage III) and TCRB^hi^ CD69^neg^ (stage IV) thymocytes derived from WT-Nu (gray) and *lamp2* KO-Nu (blue) thymus. **(C, D)** Dot plots show flow cytometry analysis of **(C)** SP4 maturation (CD24 and SELL/CD62L expression) and **(D)** T regulatory cell differentiation (IL2RA/CD25 and FOXP3 expression) in CD4 SP cells (CD4^+^TCRB^+^). Graphs represent the mean ± SEM of the frequency and cellularity of indicated subsets. * *p* < 0.05, ** *p* < 0.01.

**Figure S3.** T cell development in *b2m* KO and *lamp2* *b2m* dKO thymus. **(A)** CD4/CD8 expression within stages II (TCRB^int^ CD69^int^), III (TCRB^hi^ CD69^hi^) and IV (TCRB^hi^ CD69^neg^) is defined on total thymocytes. Bar graphs represent mean ± SEM absolute numbers of the indicated thymocyte subsets in *b2m* KO-WT (gray) and *lamp2* *b2m* dKO-WT (blue) ectopic thymi. * P < 0.05. **(B)** Dot plots show representative data of TCRB and IKZF2/HELIOS expression within DP (TCRB^lo^ IL2RA/CD25^-^ FOXP3^-^) and SP4 (TCRB^hi^ IL2RA/CD25^-^ FOXP3^-^) in *b2m* KO-WT and *lamp2* *b2m* dKO-WT ectopic thymi.

**Figure S4.** Analysis of MHC II processing and autophagy in *lamp2* KO TECs. Flow cytometry analysis of (**A**) hematopoietic (PTPRC/CD45^+^ EPCAM^-^), non-TEC stromal (PTPRC/CD45^-^EPCAM^-^) and TECs (PTPRC/CD45^-^EPCAM^+^) of WT (gray) and *lamp2* KO (blue) RFP-GFP-LC3 mice. (**B**) TECs isolated from WT and *lamp2* KO RFP-GFP-LC3 mice. Dot plot represents GFP and RFP expression on mTECs. (**C**) TECs from 12 days old WT and *lamp2* KO mice. Histograms show representative MHC-II (I-A^b^) and MHC-I (H2-K^b^ and H2-D^b^) staining in cell surface of cTEC (ENPEP/Ly51^+^UEA-1^-^), mTEC^low^ (ENPEP/Ly51^-^UEA-1^+^CD80^low^) and mTEC^hi^ (ENPEP/Ly51^-^UEA-1^+^CD80^high^) in WT (gray) and *lamp2* KO (blue) mice. Numbers represent the MFI (mean fluorescent intensity) in the indicated subsets. Data shown in **A** represents an average of 2 independent experiments with 5 WT and 6 *lamp2* KO thymus. (**D**) LAMP2 expression in TECs from 12 days old *lamp2* WT/KO heterozygous mice. Dot plots represent CD80/UEA-1 analysis in WT and *lamp2* KO TECs from *lamp2* WT/KO heterozygous thymus (n=6). (**E**) Histograms show representative assessments of CTSL activity in cTECs (green) and mTECs (red) from WT thymus (Top), and histograms show cTECs from WT (gray) and *lamp2* KO (bottom), as measured by the usage of Magic Red cathepsin L detection kit. (**F**) Histograms show representative assessments of lysosomal activity in WT (gray) and *lamp2* KO (blue) cTECs. (**G**) Postnatal day 3-5 WT thymus were treated with Baf-A1 (0.5 μM) for 5 h. Histograms show the representative staining with 15G4 staining in control (light gray) and Baf-A1-treated (black) in WT mTECs. Graphs represent the MFI in control (gray) and treated (dark black) mTEC (n= 3-4 animals). Data shown in (**B-E**) represents an average of 2 independent experiments with 4 WT and 7 *lamp2* KO thymus. Graphs represent mean ± SEM frequency of TEC subsets. * *P* < 0.05; ** *P* < 0.01;

**Figure S5.** Analysis of CD4^+^ TCR repertoire in *lamp2* KO thymus. (**A**) Dot plots show flow cytometry analysis of CD4 and CD8 expression on total thymocytes from WT (WT-TCR:Vβ6^TG^) and *lamp2* KO (*lamp2* KO-TCR:Vβ6^TG^) ectopic thymus transplanted under the kidney capsule of TCR:Vβ6^TG^ transgenic mice. Bar graphs are average absolute numbers of the indicated thymocyte subsets in control (gray) and mutant (blue) ectopic thymus. **(B)** Table shows the total number of sequences, total and unique (before and after normalization), TCRA clonotypes in SP4 thymocytes generated in WT (n=3) and *lamp2* KO (n=3) ectopic thymus. **(C)** Clonality index of WT- and *lamp2* KO-derived SP4 cells. Results are shown as mean ± SEM. * *P* < 0.05; ** *P* < 0.01;

**Figure S6.** Analysis of peripheral T cell compartment and immune response of WT- and *lamp2* KO-Nude mice. (**A**) Sorted and CFSE-labeled CD4^+^ and CD8^+^ T cells were stimulated *in vitro* with anti-CD3/anti-CD28 for 3 days. Histograms show representative experiment. Graph represents the precursor frequency of dividing T cells. Results are presented as mean ± SEM of 2 independent experiments (n=4). (**B**) Scheme of recall immune response protocol with *Lm.* Recipient mice were immunized with 2.10^3^ colony forming units (CFU) of *Lm* at 12 weeks post thymic transplantation and then rechallenged with 1.10^6^ CFU of *Lm* 4 weeks later. (**C**) Survival curve of Nude (n=3), WT-Nu (n=7) and *lamp2* KO -Nu (n=7). (**D**) Analysis of peripheral T cells in spleens from infected WT- Nu and *lamp2* KO-Nu mice. Graphs (left) represent the numbers of TCRB^+^ T cells in the spleen. Representative dot plots show CD44 and SELL/CD62L expression within CD4^+^ and CD8^+^ T cells. Graphs (right) show average cellularity of naïve (SELL/CD62L^+^CD44^lo^) and effector/memory (SELL/CD62L^+/-^CD44^hi^) CD4^+^ and CD8^+^ T cells. **(E**) Bacterial CFUs in the liver were determined by plating serial dilutions of organ homogenates onto BHI agar. Graphs represent the correlation between CFU counts in the liver and the percentage of IFNG producing CD4 and CD8 T cells in individual WT-Nu and *lamp2* KO-Nu mice. Data are representative of an experiment with 7 mice per group. Results are shown as mean ± SEM.
